# Supplementary figures and images for: Development of an Australian Bread Wheat Nested Association Mapping Population, A New Genetic Diversity Resource for Breeding under Dry and Hot Climates
Source: Int J Mol Sci. 2021 Apr 21;22(9):4348. doi: 10.3390/ijms22094348 (PMC8122485; doi:10.3390/ijms22094348)

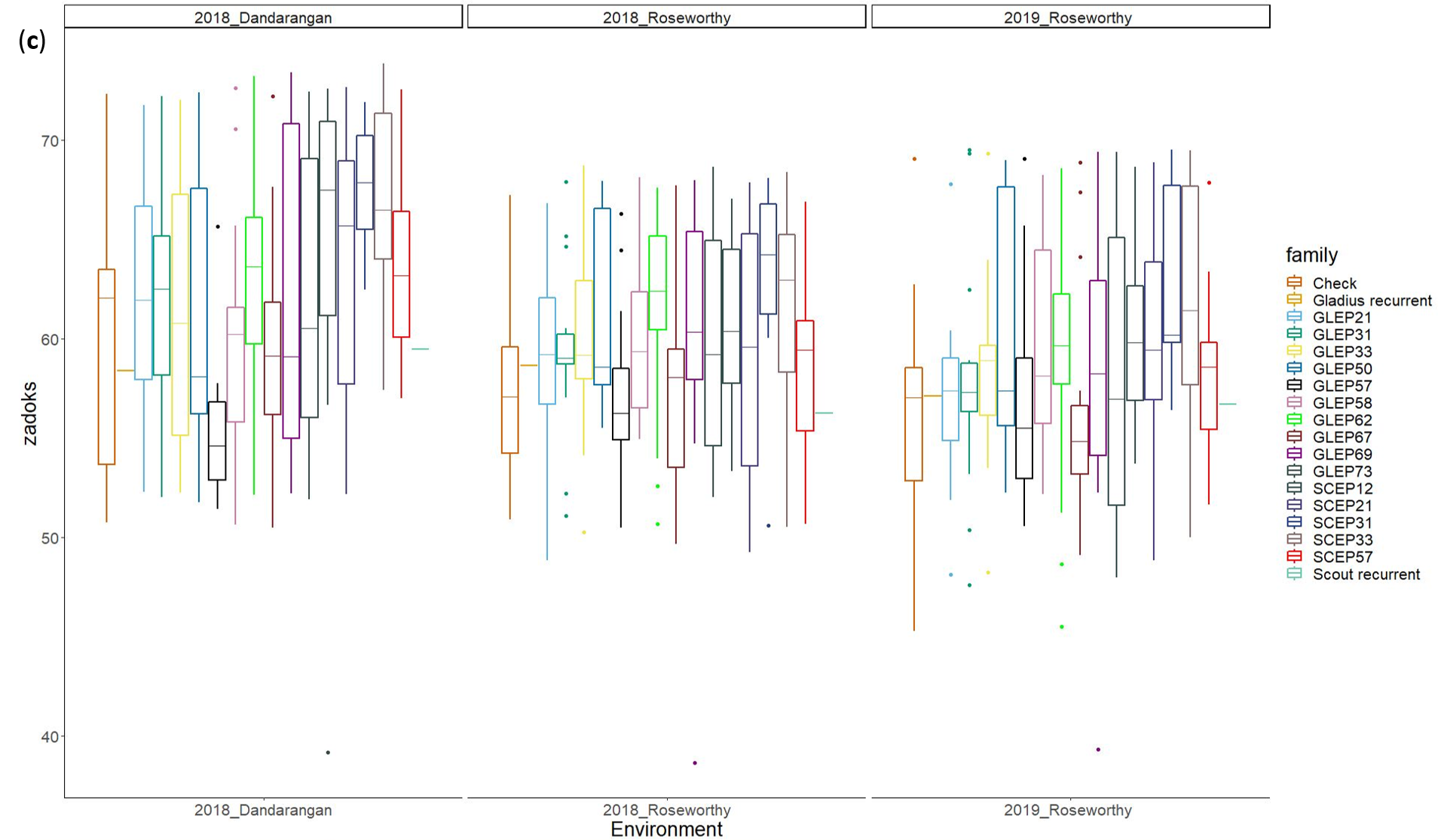

Supplement: Supplementary file 1 [file ijms-22-04348-s001.zip › Supplementary data/Figure S1-3.tif]

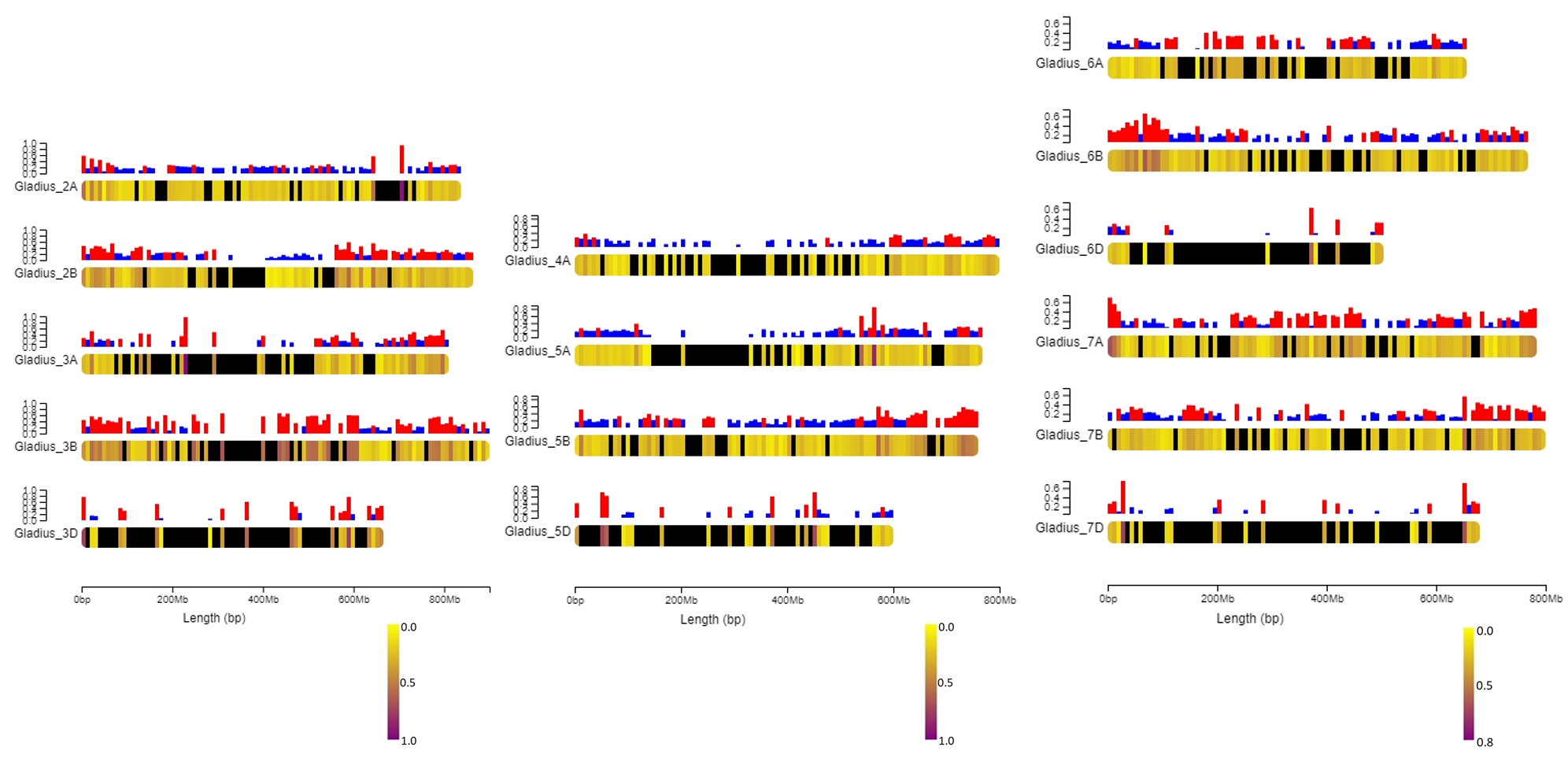

Supplement: Supplementary file 1 [file ijms-22-04348-s001.zip › Supplementary data/Figure S2.tif]

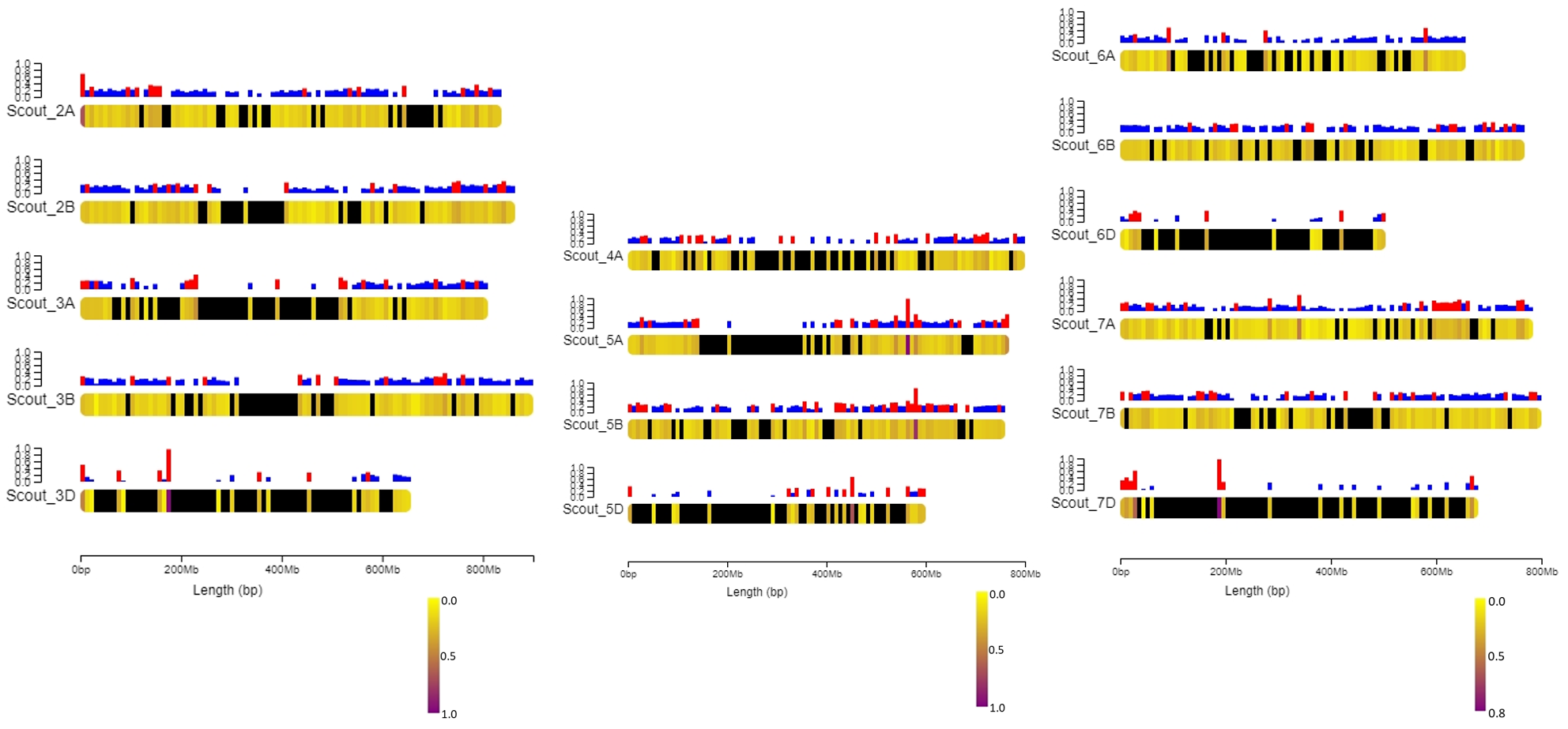

Supplement: Supplementary file 1 [file ijms-22-04348-s001.zip › Supplementary data/Figure S3.tif]

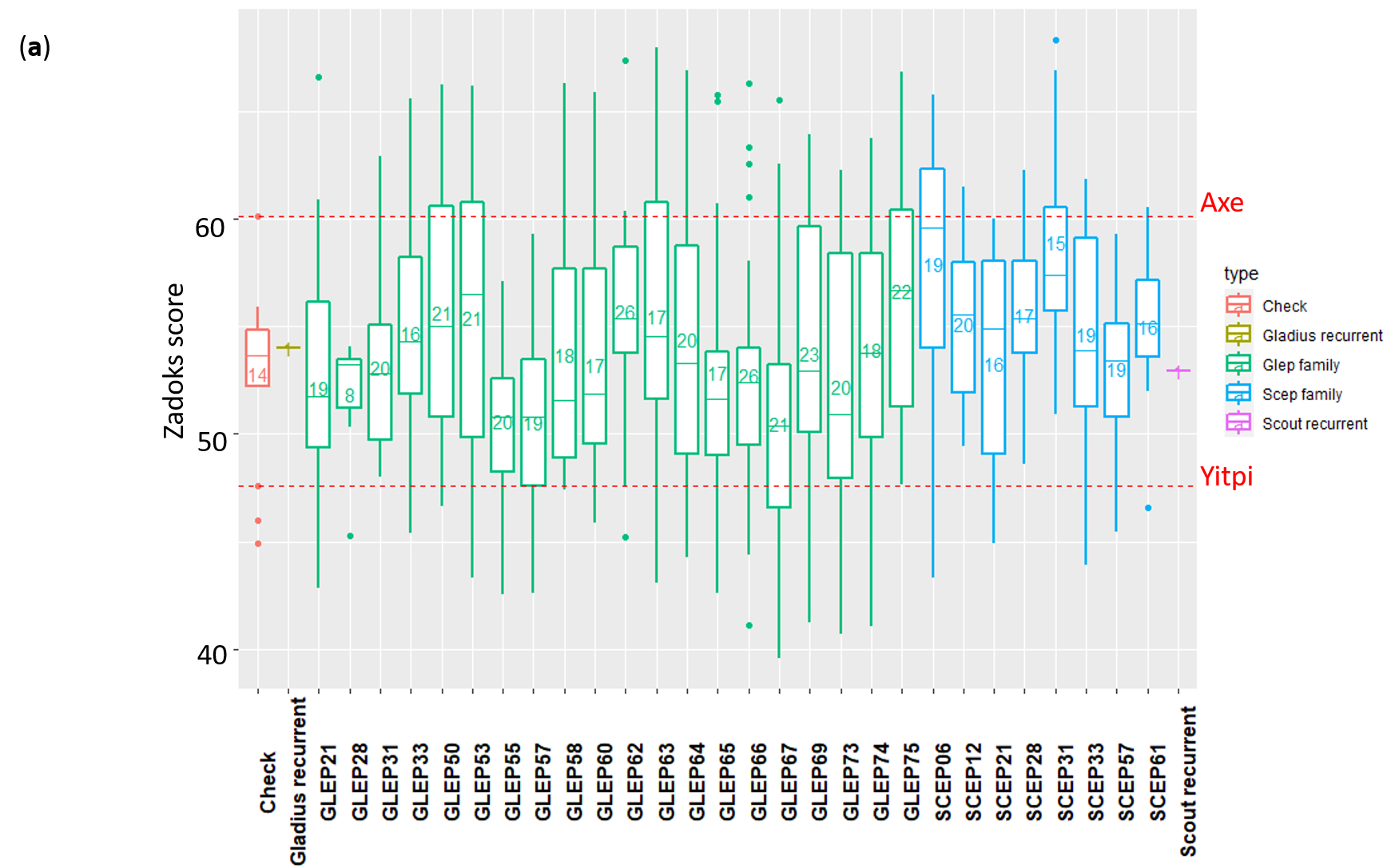

Supplement: Supplementary file 1 [file ijms-22-04348-s001.zip › Supplementary data/Figure S1-1.tif]

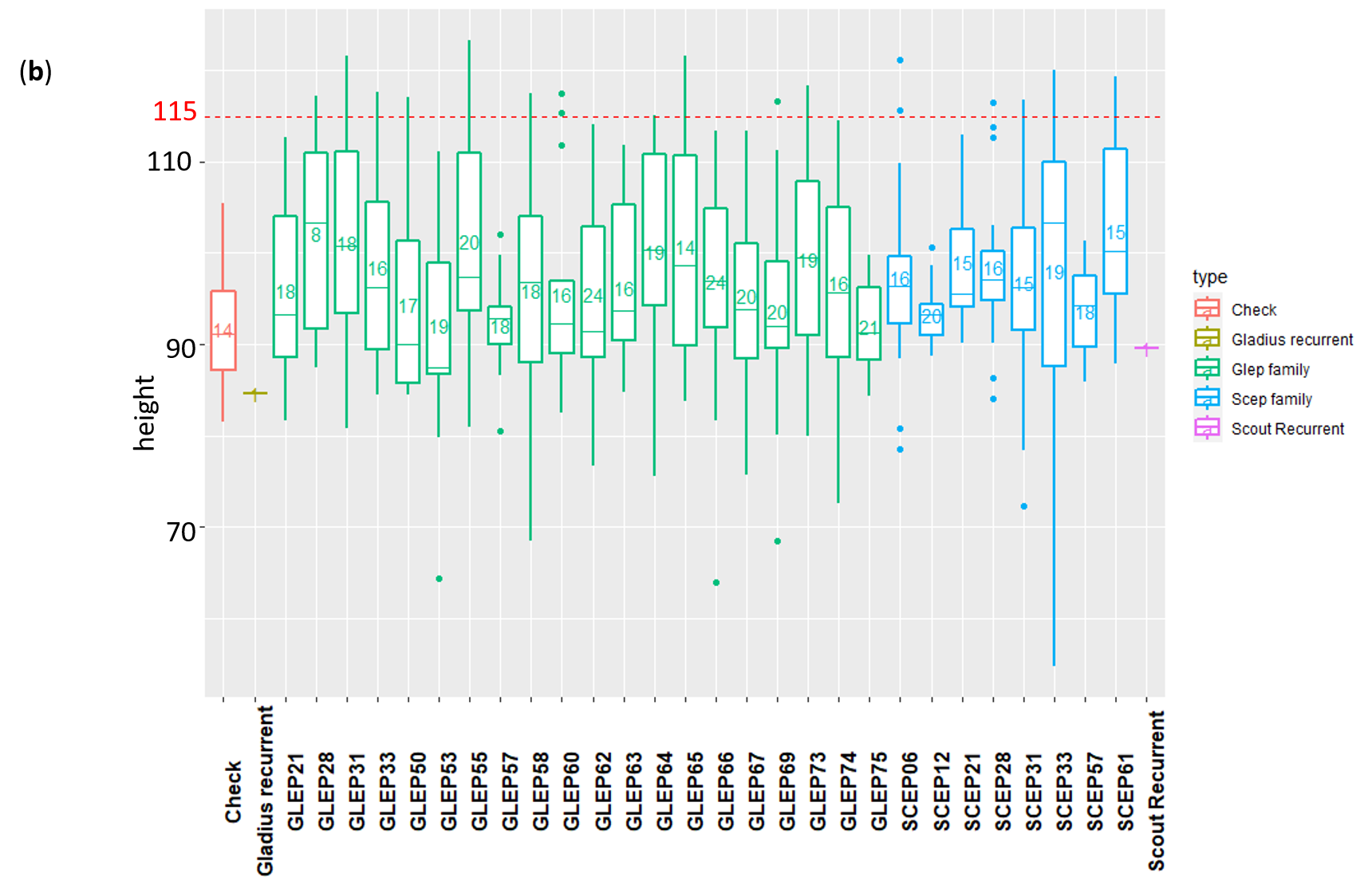

Supplement: Supplementary file 1 [file ijms-22-04348-s001.zip › Supplementary data/Figure S1-2.tif]

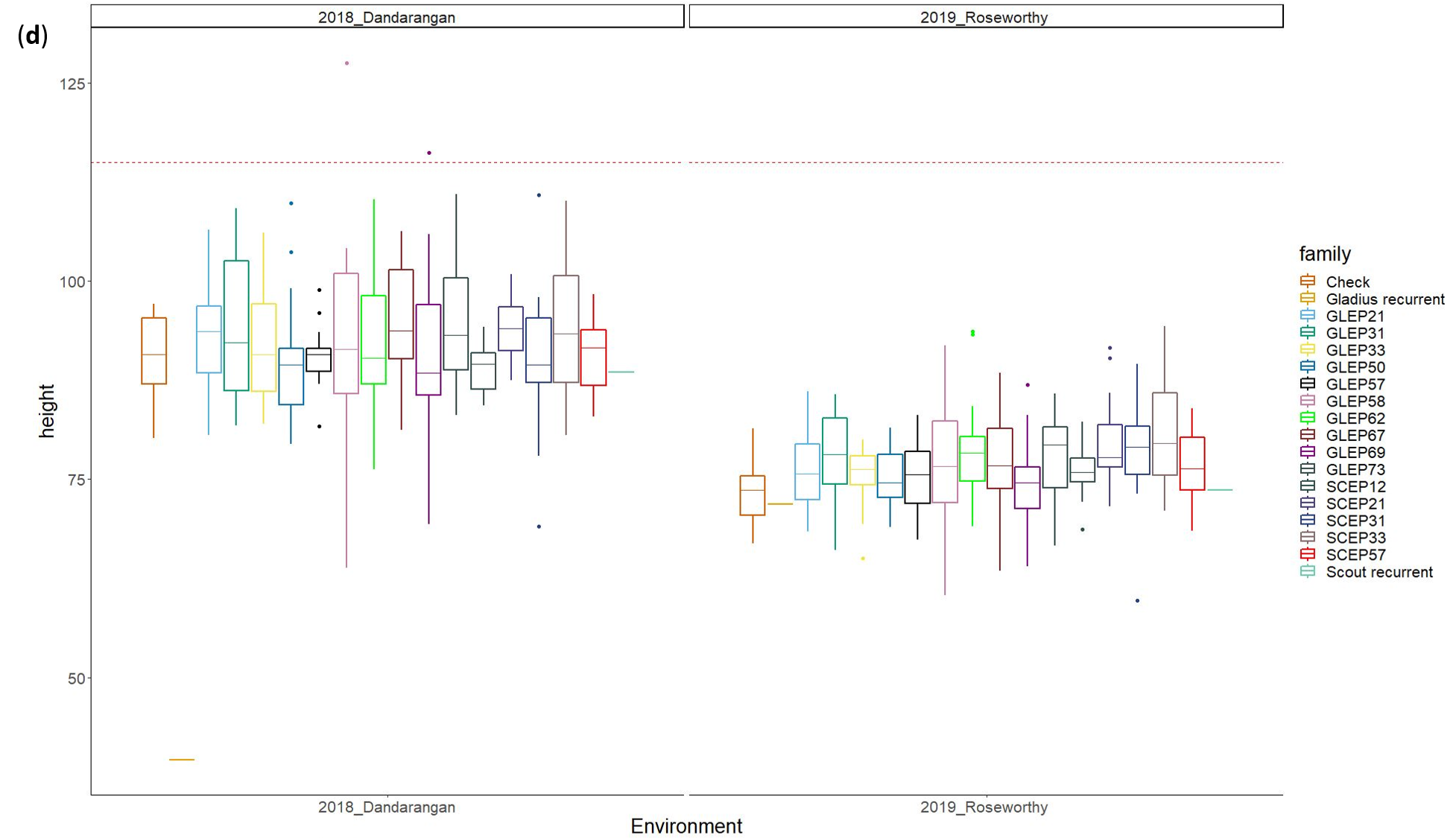

Supplement: Supplementary file 1 [file ijms-22-04348-s001.zip › Supplementary data/Figure S1-4.tif]
